# Self-reported knowledge of national guidelines for clinical screening for hip dysplasia: a web-based survey of midwives and GPs in Denmark

**DOI:** 10.3399/BJGPO.2021.0068

**Published:** 2021-07-07

**Authors:** Hans-Christen Husum, Rikke Damkjær Maimburg, Søren Kold, Janus Laust Thomsen, Ole Rahbek

**Affiliations:** 1 Department of Orthopaedics, Aalborg University Hospital, Aalborg, Denmark; 2 Department of Obstetrics and Gynaecology, Aarhus University Hospital, Aarhus, Denmark; 3 Department of Clinical Medicine, Research Unit for General Practice, Aalborg University, Aalborg, Denmark

**Keywords:** hip dislocation, congenital, mass screening, surveys and questionnaires

## Abstract

**Background:**

The positive predictive value of clinical hip examinations performed by generalist health professionals in screening for developmental dysplasia of the hip (DDH) is low and declining.

**Aim:**

To assess the self-reported recognition of nationally recommended clinical hip examinations in the screening programme for DDH in Denmark among midwives, GPs, and GPs in training.

**Design & setting:**

A cross-sectional, web-based open survey study among Danish midwives, GPs, and GPs in training.

**Method:**

Responders were asked to identify which of six written statements of clinical hip examinations were featured in the national Danish guidelines on DDH screening. Three statements were the official statements of the Ortolani, Galeazzi, and hip abduction examinations from the national guidelines, and three statements were false and constructed by the author group. Participants were asked to select up to six statements.

**Results:**

A total of 178 (58 GPs, 97 midwives, and 23 GPs in training) responses were included. Overall, 89% of responders correctly identified the Ortolani manoeuvre and 92% correctly identified one of the constructed descriptions as being false. The remaining four descriptions had significantly lower correct answer percentages ranging from 41%–58%, with significantly lower correct answer percentages of midwives for three out of all six descriptions when compared with GPs.

**Conclusion:**

The recognition of two out of three recommended clinical hip examinations in the Danish screening programme for DDH is low overall among current screeners. Efforts should be made to heighten the knowledge level by further education of screeners.

## How this fits in

The positive predictive value of clinical hip examinations made by referrers in a selective screening programme of DDH is low and declining. This study demonstrated that the self-reported knowledge among GPs and midwives of recommended clinical hip examinations in the Danish national guidelines for DDH is low. These findings could partly explain the low predictive value of clinical screening and should prompt efforts to heighten the knowledge of screeners.

## Introduction

### Background

**Figure 1.**
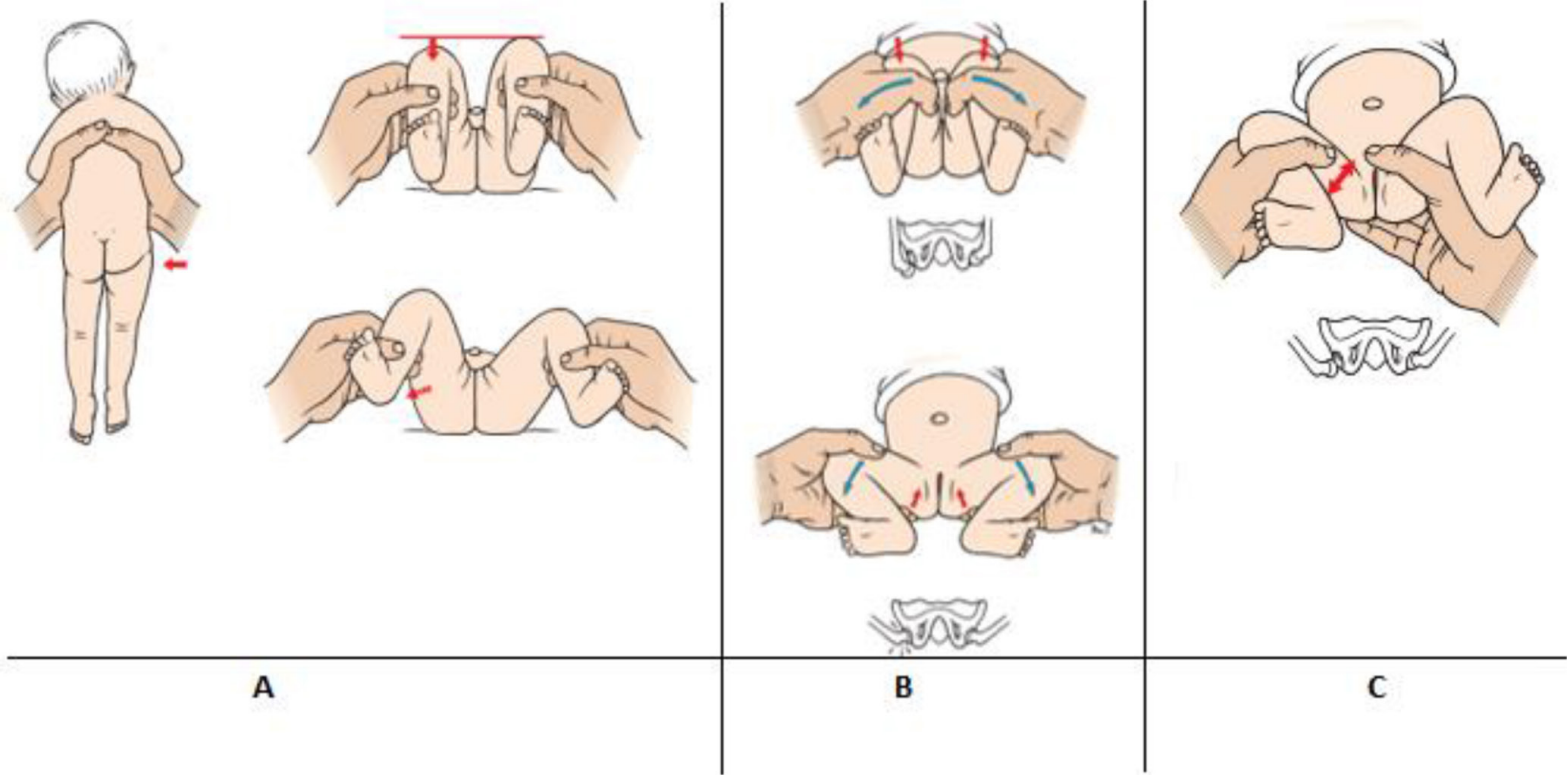
Recommended clinical hip examinations from the Danish Health Authority guidelines on screening for developmental dysplasia of the hip. A) Galeazzi test and examination of hip abduction; B) Ortolani’s manoeuvre; and C) Barlow’s manoeuvre. Images borrowed with permission from Ortopædisk Kirurgi (7th edition), FADL’s Forlag, illustrations by Birgitte Lerche.

DDH is a disorder describing abnormalities of the hip from mild underdevelopment of the acetabulum to severe deformation of the proximal femur and dislocation of the hip joint. DDH is the most common orthopaedic disorder in neonates with 0.7%–1% of all newborn children affected each year.^1,2^ A prerequisite for a screening programme to be effective is sufficient training and education of the healthcare professionals to ensure sufficient knowledge and clinical skills to perform a valid test. In Denmark, a universal clinical screening programme for DDH is implemented nationally based on guidelines made by the Danish Health Authority.^3^ The guidelines recommend a clinical hip examination is conducted shortly after birth by the midwife and a repeat examination by the GP at the 5-week postpartum appointment. The national recommendations for clinical examinations for neonatal DDH screening in Denmark follow international standards,^4,5^ which includes the Barlow and Ortolani manoeuvres, the Galeazzi test, and an examination of limited hip abduction (Figure 1). There are no formalised clinical education programmes for educating midwives and GPs to perform DDH tests, which are currently being taught as part of the non-formalised apprenticeship for midwives and GPs.

Knowledge and quality of primary screeners’ paediatric hip examinations is of paramount importance in a screening programme based on universal clinical hip examination.^6^ The positive predictive value of clinical examinations made by referrers in the UK universal clinical DDH screening programme is as low as 4%,^7^ which may explain an earlier report of the common observation of unwarranted DDH referrals for paediatric orthopaedic consultations.^8^ To the authors' knowledge, no studies have examined the self-reported knowledge of DDH screening guidelines of midwives and GPs.

### Objectives

The purpose of the present study was to assess the self-reported recognition among GPs, midwives, and GPs in training of written statements of nationally recommended clinical hip examination techniques, which are recommended in the universal clinical screening programme for DDH in Denmark.

## Method

### Study design

This was a cross-sectional, web-based open survey study. The Checklist for Reporting Results of Internet E-Surveys (CHERRIES) guideline was followed for reporting web-based surveys.^9^ Completed questionnaires were saved directly into an online database using online-survey functionality from REDCap electronic data capture tools, which was hosted at Aalborg University Hospital (AAUH), Denmark.

### Data protection

Study data were stored in REDCap, an online General Data Protection Regulation (GDPR) certified database. The first author had exclusive access to the survey data.

### Recruitment process

Email invitations were sent to GPs in the North and Central Denmark region. The GPs were asked to participate in the survey and would be financially compensated for time spent on participation with an agreed on rate with the Centre for General Practice at AAUH. As initial response rates were low, invitations were later advertised in closed social media forums for midwives and physicians.

Responders recruited through social media advertising were given no financial incentives to participate. The recruitment period ran from June 2020–September 2020.

### Participants

Inclusion criteria were as follows: certified midwife, certified GP, GP in training (no restrictions on years of training), and currently employed at a hospital or general practice in one of the five Danish geographical mainland regions, with independent health governance.

Incomplete questionnaires and GPs or midwives with previous orthopaedic or paediatric training were excluded.

### Questionnaire development

The questionnaire was designed based on a literature review and a review of the national recommendations on DDH screening.^3^ It was reviewed for validity by the author group and edited for clarification, usability, and technical functionality after pilot testing by a group of three paediatric orthopaedic surgeons and two GPs.

The questionnaire started with a header describing the purpose of the study, as well as instructions to the responders. Responders were instructed to only complete the questionnaire once. Checks of IP addresses and cookies were not used to prevent multiple entries.

The first part of the questionnaire included questions on baseline characteristics such as education and employment demographics. For the purpose of this study, responders were asked to identify which of six statements describing clinical hip examinations were featured in the national Danish guidelines on DDH screening. Three statements were the official descriptions of the Ortolani, Galeazzi, and hip abduction examinations from the national guidelines, and three statements were false and constructed by the author group (Table 1). The Barlow manoeuvre was not featured, as the illustration in the national recommendations on the Barlow manoeuvre (Figure 1) may mislead the reader to the conception that the examination should be performed in full abduction rather than mid-abduction, as Barlow himself described.^10^ Responders were asked to select up to six statements.

**Table 1.** Multiple-choice question, answer options, and interpretation in the original order. Translated to English from the original Danish

Which of these examinations are recommended in the national clinical screening programme for developmental dysplasia of the hip?
Option	Answer text	Interpretation
1	Hips and knees are bent 90 degrees, legs are spread, and the examiner examines if the hip is relocated	Correct (Ortolani)
2	Hips and knees are kept straight, during simultaneous spreading of the legs, the examiner examines the hip for a 'click'	False #1
3	Hips and knees are bent 90 degrees and the length of the thigh bone is compared	Correct (Galeazzi)
4	The examiner compares the amount of skin folds around the hips on both sides	False #2
5	Hips and knees are bent 90 degrees and the examiner examines if the hip abduction is good and symmetrical	Correct (hip abduction)
6	By moving the hip, the examiner tries to provoke an audible 'click' from the hip joint	False #3

Responders participated via a public survey link. Participants were able to edit their answers until submitting. Each participant received identical questionnaires consisting of 24 items on one page; five items were dependent on previous answers and were not visible to all participants. Answer formats ranged from text, numerical input, and multiple choice, with both single and multiple answer possibilities.

Once the questionnaire was opened, responders were instructed to provide answers with no assistance from the internet or outside help. The survey had a 30-minute time limit once opened.

All submitted questionnaires were analysed, regardless of missing answers.

### Variables

The main outcome was responders’ recognition of nationally recommended clinical hip examinations. The responses were binarised as correct or incorrect based on answers given in the questionnaire. An answer was considered correct if one of two conditions were fulfilled: 1) the statement was a correct description of a clinical hip examination featured in the national recommendations and was selected by the responder; and 2) the statement was a false description of a clinical hip examination and not selected by the responder.

### Statistical methods

Data were statistically and graphically analysed using Stata (version 16.1). Means of yearly DDH screenings were compared using one-way analysis of variance (ANOVA). All significance testing was done by comparing correct answer percentages with professions and yearly DDH screenings, by fitting a generalised linear model with identity link and robust standard errors to the binary clinical examination recognition variable. GPs were used as a reference group for comparison of correct answer percentages between professions, and the correct Galeazzi statement as reference for comparison of correct answer percentages between options. Normality of data were evaluated by inspection of QQ-plots of yearly DDH screenings.

**Figure 2.**
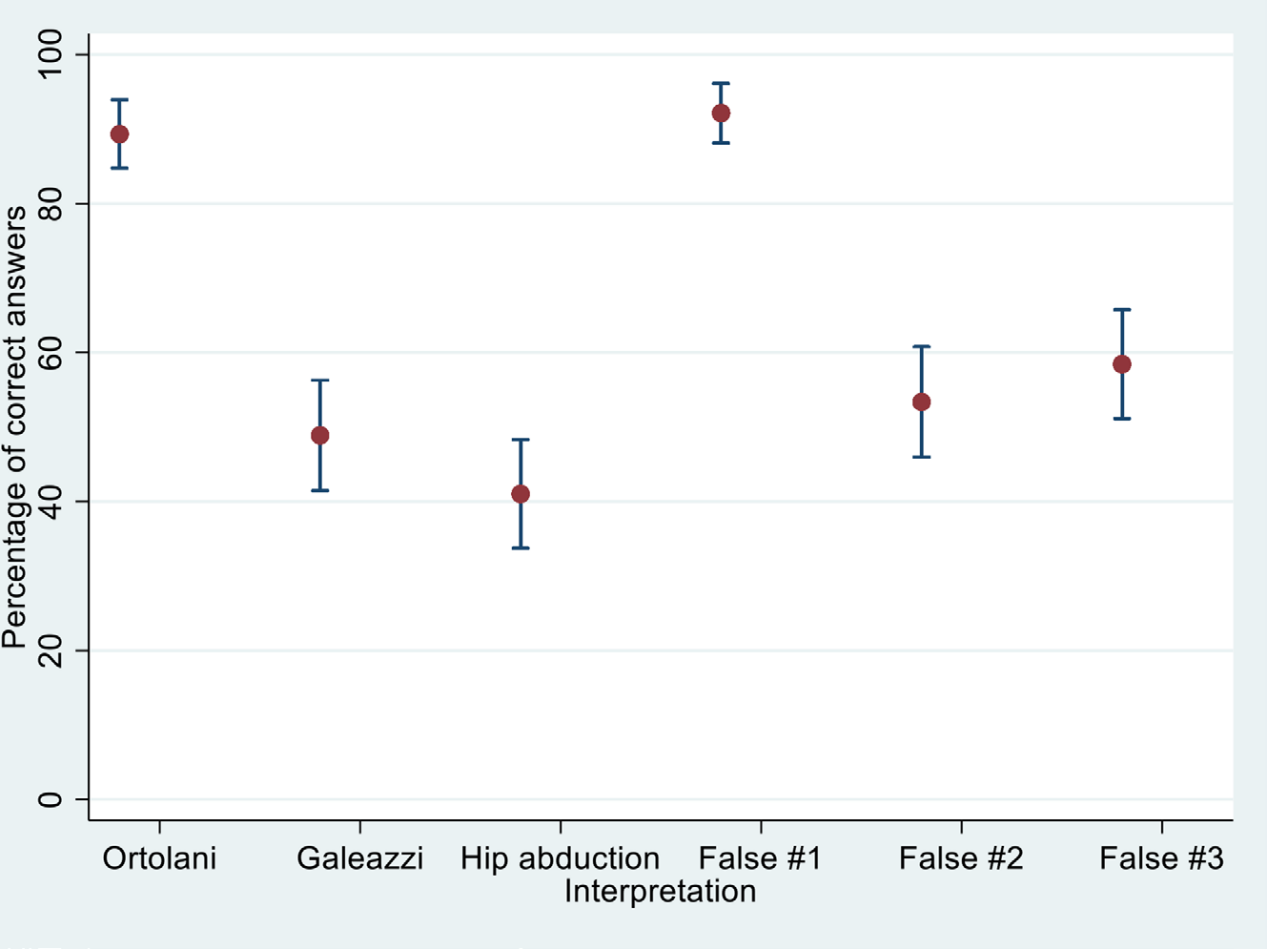
Scatter plot with 95% confidence intervals error bars, which show correct answer percentages for correct and false descriptions of nationally recommended hip examinations in developmental dysplasia of the hip screening among all responders

Results are reported as mean correct answer percentages with 95% confidence intervals (CIs) and presented graphically as a scatter plot of correct answers with error bars in the figure for correct and false answer options (Figure 2). Yearly DDH screenings are reported as means stratified by profession with given standard deviations (SDs). Statistically significant outliers (*P*<0.05) calculated based on the generalised linear model are marked as bold in Table 2.

**Table 2.** Mean correct answer percentages of correct and false statements stratified by profession of responder

Profession	*n*	Interpretation	Mean correct answer, %	95% CI
GP	58	Ortolani	84	73 to 93
Midwife	97	Ortolani	92	84 to 96
GPiT	23	Ortolani	91	72 to 99
GP	58	Galeazzi	59	45 to 71
Midwife	97	Galeazzi	**41**	**31 to 52^a^**
GPiT	23	Galeazzi	57	34 to 77
GP	58	Hip abduction	43	30 to 57
Midwife	97	Hip abduction	41	31 to 52
GPiT	23	Hip abduction	35	16 to 57
GP	58	False #1	98	91 to 100
Midwife	97	False #1	**88**	**79 to 93^a^**
GPiT	23	False #1	96	78 to 100
GP	58	False #2	50	37 to 63
Midwife	97	False #2	56	45 to 66
GPiT	23	False #2	52	31 to 73
GP	58	False #3	67	54 to 79
Midwife	97	False #3	**47**	**37 to 58^a^**
GPiT	23	False #3	83	61 to 95

Significant results are marked as bold. ^a^Significance testing done by general linear modelling with GP as the reference profession. GPiT = GP in training.

### Ethical considerations

Participants were aware that they would be compared with each other and that the anonymised results of the survey would be analysed and published.

## Results

A total of 198 responses were received. Five were not marked complete, seven did not match the inclusion criteria for profession, eight were not currently employed in one of the five Danish mainland regions, and none had received previous orthopaedic training, which left a total of 178 responses to be included in this study.

The professions of included responders were: 58 (33%) GPs, 97 (54%) midwives, and 23 (13%) GPs in training. Responders covered all five geographical areas in Denmark, with 24 (13%) responses from North Denmark Region, 54 (30%) from Central Denmark Region, 36 (20%) from Region of Southern Denmark, 25 (14%) from Region Zealand, and 39 (22%) from Capital Region of Denmark. The mean number of infants screened yearly for DDH was significantly higher for midwives at 67 a year (SD 33) when compared with GPs and GPs in training with 26 (SD 17) and 28 a year (SD 26), respectively.

Overall, 89% (95% CI = 84% to 93%) of responders identified the statement describing the Ortolani manoeuvre as correct. For one of the constructed statements, 92% (95% CI = 87% to 96%) correctly identified the statement as being false. The remaining four statements had significantly lower correct answer percentages ranging from 41%–58%, with significantly lower correct answer percentages of midwives for three out of all six statements when compared with GPs. There was no significant differences in correct answer percentages stratified by yearly DDH examinations when adjusting for profession.

Correct answer percentages are presented in Figure 2 and stratified by profession in Table 2. Nine responders (5%) correctly identified all correct examination statements and seven (4%) correctly identified all false examination statements. No responders correctly classified all correct and false answer statements.

## Discussion

### Summary

This study demonstrates that self-reported recognition of written statements of nationally recommended clinical hip examinations in the screening of DDH among GPs, midwives, and GPs in training was low (<60%) overall for two out of three correct statements of recommended examinations. Further, over 50% of responders were unable to identify two out of three statements as being false.

### Strengths and limitations

Responses were collected using an online anonymised survey as a low-cost and easily accessible tool, with the added benefit of increasing the reach of the questionnaire, as it was expanded from email invitations to include invitations via closed social media groups for health professionals. A drawback of these choices was the inability to estimate response rates for the survey invitation as the number of active users in the groups was unknown. The medical specialty of the physicians in the physician group was also unknown. As a consequence, the internal and external validity of the sample population is difficult to assess.

The identity of each participant could not be confirmed as recruitment was not made directly via personal email for all responders. The social media group for physicians used to advertise the study implements an identity check to verify that members hold a Danish medical authorisation and is a tightly moderated forum. In the social media group for midwives used to advertise the study, an administrator makes an assessment of a user’s profile before entering a new member. However, these validations do not ensure only midwives can be accepted for inclusion in the social media group and the GP specialty of the included physicians cannot be confirmed. Further, IP tracking and cookie checks were not used and, therefore, double entries were not monitored. Responders were instructed to rely on their own knowledge and not seek outside help, but no checks were made to ensure their adherence to these instructions. As midwives make up 55% of the sample, but only 34% of the background population of screeners, the study population is not an accurate representation of the background population, which should be considered when interpreting the overall correct answer percentages of this study.

Despite these limitations, participants were expected to have adhered to the instructions provided in the questionnaire, thus making their answers valid.

### Comparison with existing literature

Screening for DDH in Denmark is described in the national recommendations for neonatal DDH screening. The guidelines recommend that the neonate clinical hip examination should include the Barlow and Ortolani manoeuvres, the Galeazzi test, and a test for limited hip abduction. However, it is the authors’ experience that, in reality, generally only the Ortolani manoeuvre is performed in the clinical screening by midwives and GPs. If the national recommendations are not reflected in daily clinical practice, this could explain the high overall correct answer rate for the description of the Ortolani manoeuvre, but also the low correct answer rates for the Galeazzi and hip abduction tests.

These findings present a challenge, as clinical screeners are generally able to recognise the examination they have been taught and are using in clinical practice, but are unable to recognise recommended examinations that are, in the authors' experience, rarely used outside orthopaedic consultations. The Barlow and Ortolani manoeuvres have high negative predictive values but low positive predictive values^11^ when used exclusively; when combining these manoeuvres the sensitivity and specificity increases to 74%–99% and 98%–99%, respectively, with variation depending on the examiner’s skill level.^12^ Reliance on a single clinical hip examination is, therefore, inadvisable as it decreases the predictive value of the screening.

The positive predictive value of clinical screening in referrals in the UK, where a screening programme for DDH similar to the Danish screening is implemented, is low, and has been steadily declining over the past 20 years. It has declined from a positive predictive value of clinical examinations of referrers at 28% in 1997 to 4% in 2015. This has been attributed to an expansion and fragmentation of the pool of screeners.^7,13^ Düppe and Danielsson found a correlation between delegating clinical hip examinations to screeners not involved in the treatment or specialised diagnostics of DDH and an increase in referral and treatment rates without lowering the rate of late diagnosis of DDH in newborns.^1^

There exists no formalised training in DDH screening for midwives and GPs in Denmark, even though all Danish midwives and GPs take part in the primary clinical screening, and it has been established that clinical hip examinations are difficult to perform and interpret correctly, even for hip specialists.^14^

The aforementioned British and Swedish studies^1,7,13^ conclude that including professions not specialised in paediatric hip diseases lowers the efficacy of a DDH screening programme. The present survey suggests that a possible explanation could be that the training of clinical screeners may be inadequate in giving screeners the necessary knowledge of which hip examinations to perform, which lowers their chances of a true positive examination result.

Further, the Danish screening for DDH is not centralised to examiners with specialist training in hip examination and treatment. Similar to the programme in the UK, it is performed by generalist health professionals. The number of screeners, combined with the reported yearly DDH screenings from responders in this study, means that with an underlying incidence rate of DDH of 0.7%–1%,^1,2^ an examiner in the Danish screening programme for DDH will rarely experience a positive hip examination. Additionally, if a screener detects a positive result, contrary to hip specialists, an immediate follow-up ultrasound examination is not available and validation of a positive clinical result is, therefore, not possible.

### Implications for practice

These findings provide a possible partial explanation for the observed low predictive value of clinical hip examinations performed by primary DDH screeners. They suggest a need for efforts to be made to revise the training of DDH screeners in Denmark in order to increase their level of knowledge.

The recognition of written descriptions of two out of three recommended clinical hip examinations in the Danish screening programme for DDH is overall low among current screeners. Screeners need further education in order to increase the efficacy of clinical screening for DDH.
